# Correction: Frequency of co-seropositivities for certain pathogens and their relationship with clinical and histopathological changes and parasite load in dogs infected with *Leishmania infantum*

**DOI:** 10.1371/journal.pone.0296161

**Published:** 2023-12-15

**Authors:** Vale´ria da Costa Oliveira, Artur Augusto Velho Mendes Junior, Luiz Claudio Ferreira, Tatiana Machado Quinates Calvet, Shanna Araujo dos Santos, Fabiano Borges Figueiredo, Monique Paiva Campos, Francisco das Chagas de Carvalho Rodrigues, Raquel de Vasconcellos Carvalhaes de Oliveira, Elba Regina Sampaio de Lemos, Tatiana Rozental, Raphael Gomes da Silva, Maria Regina Reis Amendoeira, Rayane Teles-de-Freitas, Rafaela Vieira Bruno, Fernanda Nazare Morgado, Luciana de Freitas Campos Miranda, Rodrigo Caldas Menezes

The fourth author’s name is spelled incorrectly. The correct name is: Tatiana Machado Quintaes Calvet. The correct citation is: Oliveira VdC, Junior AAVM, Ferreira LC, Calvet TMQ, dos Santos SA, Figueiredo FB, et al. (2021) Frequency of co-seropositivities for certain pathogens and their relationship with clinical and histopathological changes and parasite load in dogs infected with Leishmania infantum. PLoS ONE 16(3): e0247560. https://doi.org/10.1371/journal.pone.0247560

There is an error in Table 2. The histological change in the Sample “Mammary gland (n = 28)” should be “Non-granulomatous mastitis”, not “Non-granulomatous endocarditis”.

**Table 2:** Main histological changes in dogs infected with *Leishmania infantum*, August 2016 to January 2019 (Barra Mansa, Rio de Janeiro, Brazil).

Sample	Histological changes	Monoinfected (n = 16)	Co-seropositive (n = 50)	p-value^a^
Skin (n = 66)	Granulomatous dermatitis	12 (75.0%)	34 (68.0%)	0.758
	Non-granulomatous dermatitis	0 (0.0%)	6 (12.0%)	-^d^
	Hyperkeratosis	10 (62.5%)	28 (56.0%)	1.000
	Total	14 (87.5%)	45 (90.0%)	0.204
Spleen (n = 66)	Granulomatous splenitis	7 (43.7%)	37(74.0%)	0.068
	Pyogranulomatous splenitis	2 (12.5%)	2 (4.0%)	0.245
	Non-granulomatous splenitis	1 (6.2%)	2 (4.0%)	1.000
	Total	10 (62.5%)	41 (82.0%)	0.019^c^
Liver (n = 66)	Granulomatous hepatitis	12 (75.0%)	39 (78.0%)	1.000
	Pyogranulomatous hepatitis	2 (12.5%)	1 (2.0%)	0.143
	Non-granulomatous hepatitis	2 (12.5%)	5 (10.0%)	1.000
	Vacuolar degeneration of hepatocytes	9 (56.2%)	12 (24.0%)	0.068
	Total	16 (100.0%)	46 (92.0%)	1.000
Lung (n = 66)	Granulomatous pneumonia	3 (18.7%)	22 (44.0%)	0.083
	Pyogranulomatous pneumonia	3 (18.7%)	7 (14.0%)	0.695
	Non-granulomatous pneumonia	2 (12.5%)	6 (12.0%)	1.000
	Total	8 (50.0%)	35 (70.0%)	0.538
Tricuspid (n = 66)	Granulomatous endocarditis	2 (12.5%)	7 (14.0%)	1.000
	Non-granulomatous endocarditis	1 (6.2%)	2 (4.0%)	1.000
	Total	3 (18.7%)	9 (18.0%)	1.000
Mitral (n = 66)	Granulomatous endocarditis	2 (12.5%)	3 (6.0%)	0.588
	Pyogranulomatous endocarditis	2 (12.5%)	0 (0.0%)	-^d^
	Non-granulomatous endocarditis	2 (12.5%)	2 (4.0%)	0.245
	Total	6 (37.5%)	5 (10.0%)	0.019^c^
Mammary gland^b^ (n = 28)	Granulomatous mastitis	4 (44.4%)	9 (47.4%)	0.719
	Non-granulomatous mastitis	0 (0.0%)	5 (26.3%)	-^d^
	Total	4 (44.4%)	14 (73.6%)	1.000
Uterus^b^ (n = 28)	Granulomatous metritis	0 (0.0%)	4 (21.0%)	-^d^
	Pyogranulomatous metritis	0 (0.0%)	1 (5.3%)	-^d^
	Non-granulomatous metritis	0 (0.0%)	1 (5.3%)	-^d^
	Total	0 (0.0%)	6 (31.6%)	-^d^

n: number of dogs. Total: total number of dogs with histological changes in the organ. Co-seropositive: *L*. *infantum*-seropositive dogs with confirmed infection with this parasite by qPCR and/or culture and with antibodies against at least one pathogen other than *L*. *infantum*. Monoinfected: *L*. *infantum*-seropositive dogs with confirmed infection with this parasite by qPCR and/or culture and without antibodies against certain pathogens other than *L*. *infantum*.

^a^ The p-values were calculated using Fisher’s exact test (p ≤ 0.05).

^b^ The frequencies were calculated and statistical analysis was performed in 9 dogs of the monoinfected group and in 19 dogs of the co-seropositive group.

^c^ Statistically significant difference (p ≤ 0.05).

^d^ Statistical analysis was not possible because of the absence of certain histological changes in one of the groups.
