# Supplementary material for: Frequency of co-seropositivities for certain pathogens and their relationship with clinical and histopathological changes and parasite load in dogs infected with Leishmania infantum
Source: PLoS One. 2021 Mar 11;16(3):e0247560. doi: 10.1371/journal.pone.0247560 (PMC7951870; doi:10.1371/journal.pone.0247560)
Supplement: S1 Table — (DOC) [file pone.0247560.s001.doc]

**S1 Table. Median number of inflammatory cells observed in each organ of dogs infected with *Leishmania infantum* according to the co-seropositivity for certain pathogens, August 2016 to January 2019 (Barra Mansa, state of Rio de Janeiro, Brazil).**

| Samples | Number of inflammatory cells/mm^2^ | | | | | | | | | | | | | | |
| --- | --- | --- | --- | --- | --- | --- | --- | --- | --- | --- | --- | --- | --- | --- | --- |
|  | L (n=16) | | Tg (n=19) | | E+Tg (n=12) | | E (n=7) | | E+A+Tg (n=5) | | E+A (n=3) | | A+Tg (n=3) | | A (n=1) ^a^ |
|  | Med | Range | Med | Range | Med | Range | Med | Range | Med | Range | Med | Range | Med | Range | Total^b^ |
| Skin (n=66) | 195 | 0- 481 | 144 | 0-587 | 67 | 0-176 | 80 | 0-367 | 172 | 112-315 | 181 | 57-346 | 89 | 0-200 | 467 |
| Spleen (n=66) | 410 | 0-707 | 467 | 0-702 | 592 | 417-733 | 490 | 0-663 | 601 | 412-753 | 335 | 0-517 | 505 | 0-556 | 625 |
| Liver (n=66) | 197 | 0-534 | 295 | 0-936 | 298 | 0-548 | 396 | 94-491 | 288 | 83-342 | 389 | 229-465 | 211 | 0-381 | 184 |
| Lung (n=66) | 103 | 0-434 | 360 | 0-602 | 233 | 0-474 | 439 | 0-618 | 400 | 0-595 | 294 | 202-508 | 414 | 0-500 | 294 |
| Tricuspid (n=66) | 0 | 0-686 | 0 | 0-270 | 0 | 0-104 | 0 | 0-107 | 0 | 0-251 | 0 | 0-0 | 0 | 0-0 | 300 |
| Mitral (n=66) | 0 | 0-471 | 0 | 0-371 | 0 | 0-339 | 0 | 0-0 | 0 | 0-101 | 0 | 0-113 | 0 | 0-0 | 174 |
| Uterus (n=28) | 0 | 0-0 | 0 | 0-359 | 0 | 0-320 | 0 | 0-151 | 27.5 | 0-55 | 0 | 0-415 | 272 | 0-544 | -^c^ |
| Mammary gland (n=28) | 149 | 67-267 | 449.5 | 392-493 | 291 | 0-581 | 302 | 57-435 | 50 | 0-100 | 367 | 323-405 | 374 | 234-514 | -^c^ |

Med: median; L: *L. infantum*; Tg: *Toxoplasma gondii*; E: *Ehrlichia* spp.; A: *Anaplasma* spp*.*

^a^ Group composed of one dog (male).

^b^ The total number of inflammatory cells/mm^2^ was calculated, as only one male dog was positive.

^c^ Organ not analyzed.
